# Optimization of the irradiation dose for *Anopheles coluzzii* for implementation of the sterile insect technique

**DOI:** 10.1186/s13071-026-07396-z

**Published:** 2026-05-05

**Authors:** Bouraïma Vincent Séré, Simon Péguédwindé Sawadogo, Ablawa Prudenciène Agboho, Bèwadéyir Serge Poda, Dekonone Jean Jacques Tioyé, Zegué Souleïmane Ouattara, Judicael Ouedraogo, Tarwendpanga Francois-Xavier Ouédraogo, Nanwintoum Séverin Bimbilé Somda, Abdoulaye Diabaté, Olivier Gnankiné, Roch Kounbobr Dabiré

**Affiliations:** 1 Institut de Recherche en Sciences de la Santé (IRSS), Direction Régionale de l’Ouest (DRO), 399 Avenue de La Liberté, 01 BP 545 Bobo-Dioulasso 01, Burkina Faso; 2 Laboratoire d’Entomologie Fondamentale Et Appliquée (LEFA), Université Joseph KI-ZERBO, 03 BP 7021 Ouagadougou, Burkina Faso; 3 Centre International de Recherche-Développement sur l’Élevage en zone Subhumide (CIRDES), 01 BP 454 Bobo-Dioulasso 01, Burkina Faso; 4 Université Nazi Boni, 01 BP 1091 Bobo-Dioulasso 01, Burkina Faso; 5 Université Nobert Zongo de Koudougou, 01 BP 376 Koudougou 01, Burkina Faso

**Keywords:** Malaria, *Anopheles coluzzii*, Sterile insect technique, Irradiation dose, Biological traits

## Abstract

**Background:**

The sterile insect technique (SIT) is a population suppression strategy that involves releasing sterile male insects, which mate with wild females, thereby inducing sterility in the target population. Although SIT represents a promising complementary approach for malaria vector control, its success depends on identifying an irradiation dose that ensures high sterility while preserving the biological performance of males. This study aimed to determine the optimal irradiation dose for sterilizing *Anopheles coluzzii*, a major malaria vector in West Africa, under laboratory conditions in Burkina Faso.

**Methods:**

Experiments were conducted using the 13th generation (G13) of a laboratory strain of *Anopheles coluzzii*. Pupae were irradiated with doses ranging from 40 to 120 Gy. Key biological parameters, including adult emergence rate, insemination rate, fertility (egg hatching rate), and survival, were evaluated under controlled laboratory conditions.

**Results:**

Irradiation did not affect adult emergence at any of the tested doses. Though insemination rates declined at higher doses, they were not significantly impacted by doses between 40 and 70 Gy. All irradiation doses negatively affected mosquito survival, with more pronounced effects observed at higher doses. Egg hatching rates remained unaffected at 40 and 50 Gy, were significantly reduced at 60 Gy, and were completely suppressed at 70 Gy.

**Conclusions:**

An irradiation dose of 70 Gy appears optimal for *Anopheles coluzzii*, because it induces complete sterility (0% hatching) while maintaining insemination and emergence rates similar to those of the control group, despite a dose-dependent reduction in survival. Further studies are needed to evaluate the mating competitiveness of males irradiated at this dose under semi-field and field conditions to support the implementation of the sterile insect technique (SIT) in Burkina Faso.

**Graphical Abstract:**

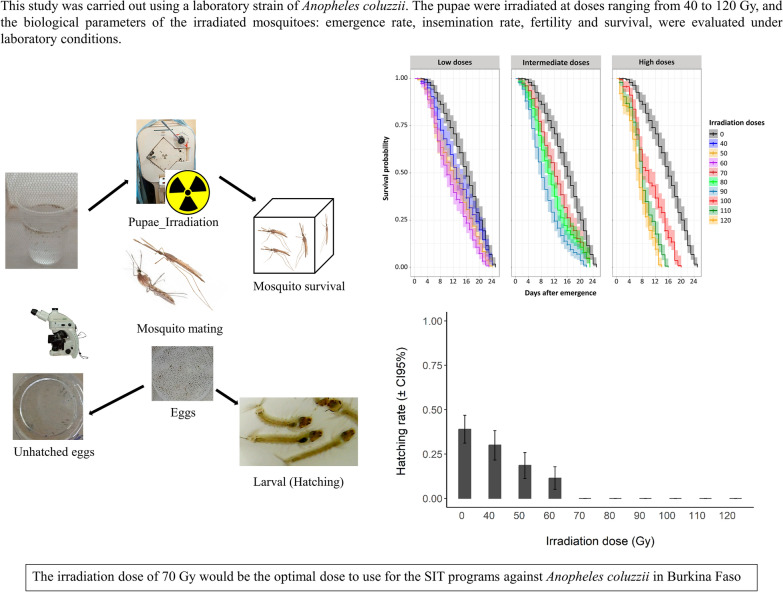

**Supplementary Information:**

The online version contains supplementary material available at 10.1186/s13071-026-07396-z.

## Background

Malaria is a life-threatening disease transmitted to humans by *Anopheles* mosquitoes [1]. In 2024, the World Health Organization (WHO) estimated that there were approximately 282 million malaria cases worldwide, resulting in about 610,000 deaths. The WHO African Region accounted for 94% of global malaria cases and 95% of the global disease burden [1].

Malaria control strategies target both the parasite and its vector, with vector control remaining a cornerstone of prevention efforts. Current approaches rely largely on insecticide-based interventions, including insecticide-treated nets (ITNs) and indoor residual spraying (IRS) [2, 3]. However, the effectiveness of these methods is increasingly compromised by the widespread development of insecticide resistance in malaria vectors [4–9]. This challenge underscores the urgent need for innovative and complementary tools to sustain and enhance malaria vector control.

In this context, novel approaches such as vaccination, genetically modified mosquitoes, and the sterile insect technique (SIT) are being explored [10–12]. The concept of SIT emerged independently in the 1940s through the work of A.S. Serebrovskii in the USSR, F.L. Vanderplank in Tanzania, and E.F. Knipling in the USA [13]. SIT involves releasing large numbers of sterile insects into an area to reduce the fertility of a wild population of the same species. It is a species-specific, non-polluting, and environmentally friendly method that aims to suppress target insect populations by reducing their reproductive capacity [13, 14]. Sterility can be induced in insects by several methods. It can be induced by gamma or X-rays, electron beams, or chemical methods [15–18]. When applied to mosquitoes, SIT involves the mass release of sterile males that compete with wild males for mating with wild females. Male sterilization by exposure to gamma rays is the most effective method for sterilizing mosquitoes. This treatment causes chromosomal damage and lethal dominant mutations in sperm, resulting in the production of infertile eggs and, consequently, a reduction in vector population [19, 20]. The sterile insect technique (SIT) has already proven effective against several disease vectors [21], including tsetse flies in Zanzibar [22], *Aedes albopictus* in Italy [23], *Anopheles albimanus* Wiedemann in El Salvador [18, 24], and *Glossina* species in Senegal [25]. Although progress has been made in irradiation techniques, attempts to apply the sterile insect technique to malaria vectors of the genus *Anopheles* have not yet yielded conclusive results [26]. Releases of chemically sterilized male *Anopheles culicifacies* or sterile hybrid males resulting from crosses between *Anopheles gambiae* s.s. and *Anopheles melas* have been unsuccessful, mainly because of reduced sexual competitiveness compared to wild males [24, 25]. Similarly, in *An. gambiae* sensu lato (s.l.), the low competitiveness of sterile males remains a major constraint, influenced by factors such as the stage of development at the time of irradiation, the type of radiation, the dose and duration of exposure, and species-specific sensitivity [28].

Furthermore, although previous work, notably that of Maïga et al. on the *An. coluzzii* strain colonized at the IPCL laboratory, explored irradiation of this species at a dose of 90 Gy, the detrimental impacts on fitness suggest that this dose exceeds the species' biological tolerance threshold. Indeed, males were unable to find females, even in the absence of direct competition [28]. It is therefore imperative to define an optimal dose specific to the local strain, capable of reconciling robust gamete sterility with competitive performance under semi-natural conditions.

Recently, several studies have provided important information on *Anopheles arabiensis*, regarding radiation dose, stage of irradiation, and competitiveness [29–31]. However, studies on insect sensitivity to radiation have revealed variations between regions and strains [32, 33]. Furthermore, specific data relating to *An. coluzzii*, one of the main malaria vectors in Africa [31], are insufficient for optimal implementation of the SIT. Yet, the success of SIT critically depends on achieving an optimal balance between induced sterility and male competitiveness. Excessive irradiation can impair survival, mating behavior, and flight ability, whereas insufficient doses may fail to induce adequate sterility [34]. This study aims to evaluate the effects of a range of irradiation doses on the main life history traits of *Anopheles coluzzii* and to identify the optimal dose that maximizes sterility while minimizing negative impacts on the mosquitoes' biological performance.

## Methods

### Mosquitoes

This study used the 13th generation *Anopheles coluzzii* strain from the Institut de Recherche en Sciences de la Santé (IRSS), Bobo-Dioulasso, Burkina Faso. At the IRSS insectarium, *An. coluzzii* larvae were reared in 1 l of demineralized water, at a density of 500 larvae per plastic container, and fed with Tetramin^®^ Baby Fish Food at increasing quantities: 0.03 g during the first 2 days, 0.06 g on days 3 and 4, and 0.09 g from day 5 onwards. Adults were maintained in 30 × 30 × 30-cm cube cages (BugDorm-1; MegaView, Taichung, Taiwan) at a density of 700 mosquitoes per cage and provided with 5% glucose solution. Females were offered rabbit blood for egg production. Rearing was conducted under standard insectary conditions (27 ± 2 ºC, 70 ± 10% relative humidity, and 12L:12D photoperiod).

### Mosquito irradiation

Pupal irradiation was performed following the protocol described by Poda [29]. To reduce the effect of irradiation on the life history traits of mosquitoes, pupae aged approximately 20 h old [30] were collected and placed in individual plastic cups (diameter: 45 mm; height: 85 mm) with 200 pupae per cup in six replicates. Replicates were performed in pairs. Each cup contained approximately 1 cm of water to maintain pupal viability while minimizing radiation attenuation. Cups were randomly assigned to either irradiated or un-irradiated (control) groups. Irradiated pupae were exposed to theoretical doses of 40, 50, 60, 70, 80, 90, 100, 110, or 120 Gy using a Gamma Cell ^137^Cs self-contained gamma irradiator at a dose rate of approximately 2.60 Gy/min. Cups were placed in the center of the irradiation chamber to ensure a homogeneous dose for the different batches of mosquitoes. Therefore, for the same dose, the two cups were introduced individually. The actual absorbed dose was verified using Gafchromic^®^ HD-V2 dosimetry film (Ashland, Bridgewater, NJ, USA) affixed to the cup walls, as described by Poda [29]. Control pupae were handled identically but were not irradiated.

### Measured biological parameters

The experiment was repeated six times. The following biological parameters were evaluated in each replicate.

#### Emergence

In each replicate, following irradiation, the 200 pupae were divided into two batches of 100 per dose and placed in clear plastic cups (diameter: 45 mm; height: 85 mm) containing 50 ml of demineralized water. These two groups were then placed in the same 30 × 30 × 30-cm cube cages (BugDorm-1; MegaView, Taichung, Taiwan). Forty-eight hours later, the number of dead pupae was counted in each cage, and the adult emergence rate was calculated as the proportion of emerged adults relative to the initial number of pupae per dose, considering the six replicates. We selected 200 pupae per dose and per replicate, assuming a sex ratio of approximately 1:1, to ensure a minimum of 60 males, 30 of which were designated for the longevity study and 30 for the fertility study.

#### Fertility

Starting at 6 a.m. the day after irradiation, the sex of the newly emerged mosquitoes was determined. Males and females were kept separately to prevent mating. Both sexes were kept under insectary conditions and provided with a 5% glucose solution for 4 to 6 days. Subsequently, 30 virgin males and 30 virgin females were randomly selected and placed together in 20 × 20 × 20-cm cages (1:1 sex ratio) for mating. Two mating combinations were tested: irradiated males with un-irradiated females and un-irradiated males with un-irradiated females (control). Mosquitoes were allowed to mate for 3 nights and were provided with a 5% glucose solution. Then, females were removed from the cages and transferred to clear plastic cups (diameter: 75 mm; height: = 100 mm); they were provided with two rabbit blood meals at 2-day intervals. Blood-fed females were maintained on a 5% glucose solution. Three days after the second blood meal, gravid females were individually placed in plastic oviposition cups (diameter: = 45 mm; height: = 85 mm) containing distilled water.

Eggs were left in water for 1 week to allow hatching. After 1 week, the hatched and unhatched eggs were counted under a binocular microscope to assess fertility.

#### Insemination

After oviposition, females were removed and dissected to extract spermathecae. Insemination status was evaluated qualitatively by the presence or absence of spermatozoa observed under a light microscope at 400 × magnification. Females that did not oviposit within 10 days were also dissected and examined using the same procedure.

#### Longevity

The day after irradiation, 30 emerged males per dose were randomly selected and placed in 20 × 20 × 20-cm cages and maintained under insectary conditions with access to a 5% glucose solution. Mortality was recorded every 24 h until all individuals had died. Dead mosquitoes were removed and counted daily.

### Statistical analysis

All statistical analyses were performed with R software (version 4.5.1) [35]. The effect of irradiation dose (10 levels: 0–120 Gy) on emergence and egg hatching was assessed using the Kruskal-Wallis test. When significant effects were detected, pairwise comparisons were performed using Dunn’s test (dunnTest function, FSA package). The effect of irradiation dose on insemination rate was analyzed using Tukey's post hoc test. Male survival was analyzed using a mixed-effects Cox proportional hazards model (*coxme* function, *coxme package*), with experimental replicate included as a random effect. Percentages are presented with their 95% confidence intervals (95% CI).

### Ethical considerations

This study constitutes a component of a larger research project, the protocol for which has been formally endorsed by the Institutional Ethics Committee of the IRSS (N/Réf. A047-2022/CEIRES).

## Results

### Adult emergence rate

**Fig. 1:**
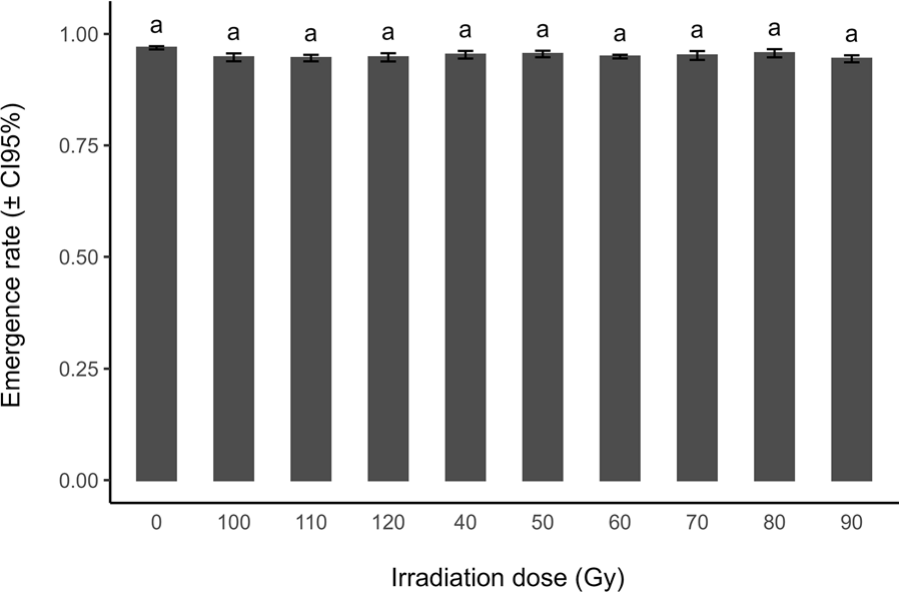
Emergence rate as a function of irradiation dose: ratio between the number of emerged adults and the total number of initial pupae per dose for the three replicates

A total of 14,400 pupae were used in the study, including 3600 in the control group and 10,800 in the irradiated group. Adult emergence rates were consistently high across all doses, ranging from 94.4% to 97.0%. No significant differences were detected between the control and irradiated groups (Kruskal-Wallis *H* test: *χ*^2^ = 13.90, *df* = 9, *P* = 0.13; Fig. 1).

### Mosquito survival

**Fig. 2:**
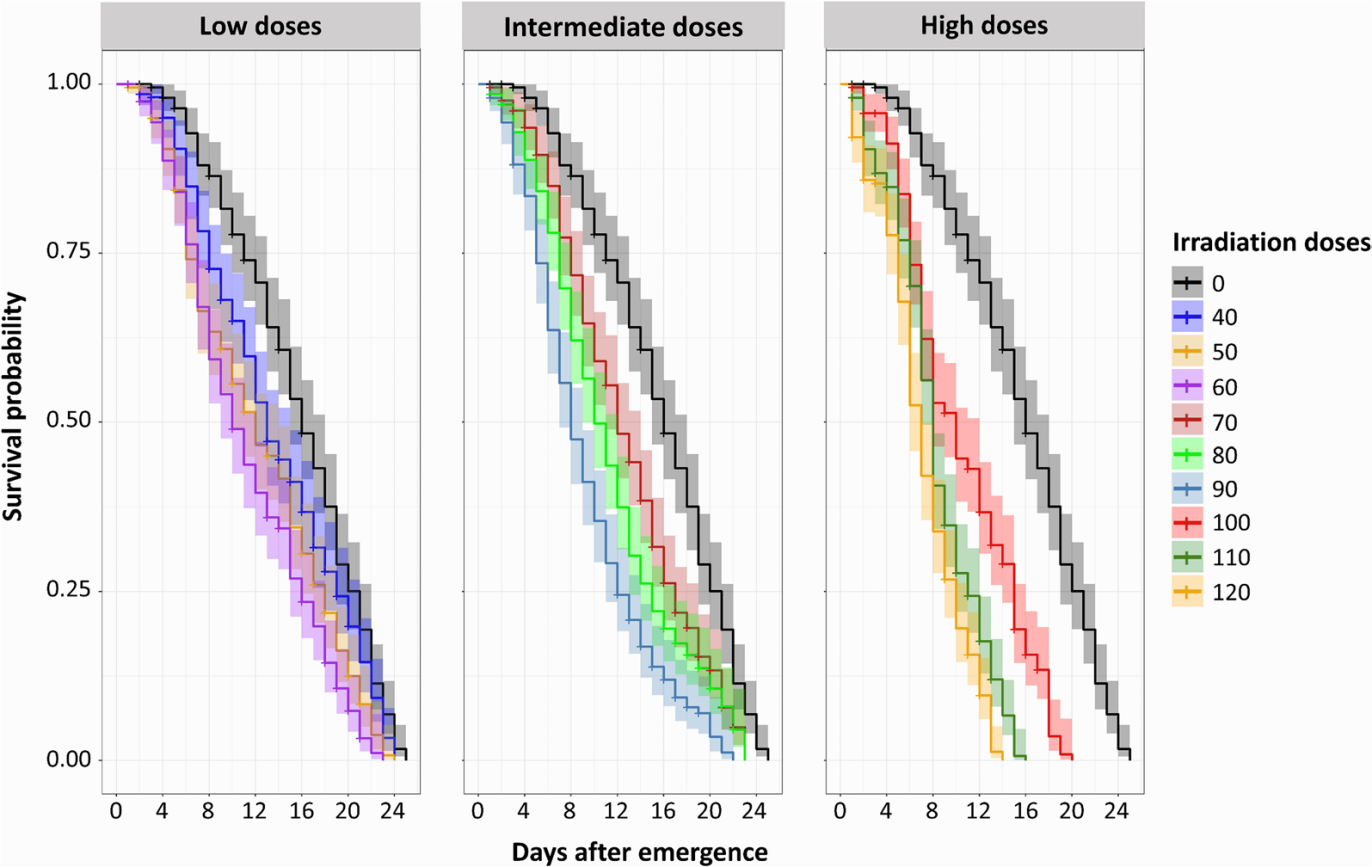
Effects of irradiation dose on mosquito survival. Survival of mosquitoes in the irradiated groups was significantly lower than in the control group (pair comparison, *P* < *0.05*)

Irradiation dose had a significant effect on mosquito survival (mixed-effects Cox model: *F* = 574.28, *P* < *0.001*; Fig. 2). Median survival times of irradiated males ranged from 8 to 13 days, compared with 16 days for control mosquitoes. While low and intermediate doses had moderate effects, higher doses (100, 110, and 120) markedly reduced maximum survival (Fig. 2, Table 1).

**Table 1:** Median survival of mosquitoes as function of irradiation dose

Irradiation doses (Gy)	Number of mosquitoes tested for the 3 repetitions (6 replicates)	Median survival [95% CI] (day)	z value	Hazard ratio (lower 0.95 to upper 0.95)	*P*–value
0	270	16 [15–17]	–	–	–
40	180	13 [12–15] *	3.27	1.33 (1.12–1.57)	0.001
50	180	12 [10–14] *	6.07	1.70 (1.43–2.02)	< 0.001
60	180	10 [9–12] *	8.46	2.11 (1.77–2.50)	< 2e-16
70	180	12 [11–14] *	5.91	1.68 (1.41–1.99)	< 0.001
80	180	10 [9–12] *	8.13	2.04 (1.72–2.42)	< 0.001
90	180	8 [7–9] *	12.90	3.16 (2.65–3.76)	< 2e-16
100	180	10 [8–12] *	10.75	2.62 (2.20–3.12)	< 2e-16
110	180	8 [7, 8] *	16.08	4.34 (3.63–5.20)	< 2e-16
120	180	7 [6, 7] *	19.07	5.82 (4.85–6.97)	< 2e-16

Star indicates statistical difference compared with the control (pairwise comparison, *P* < 0.05)

### Female insemination rate

**Fig. 3:**
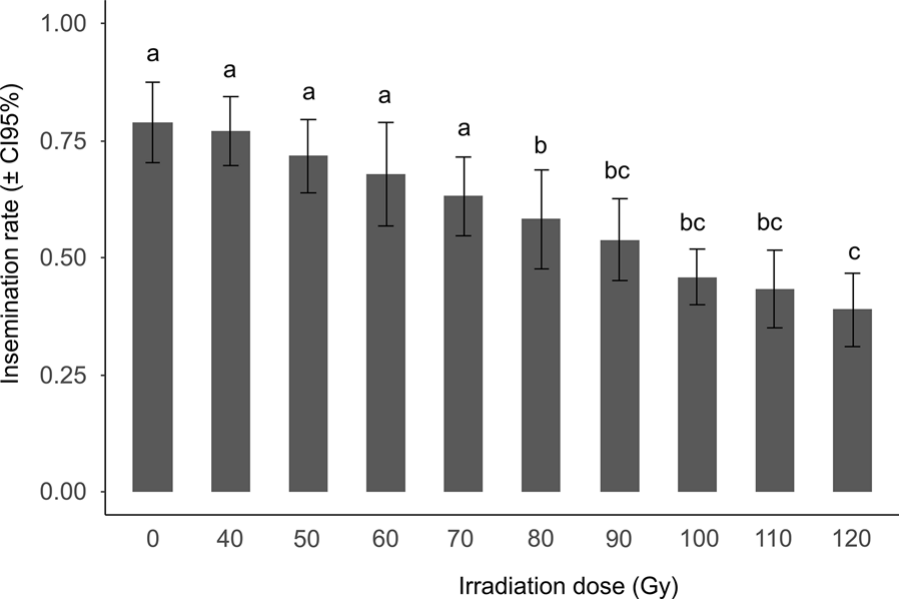
Proportion of insemination according to different irradiation doses

Female insemination rate varied significantly according to irradiation dose (*F* = 16.53, *P* < *0.001*, Fig. 3). Pairwise comparison with the control group (0 Gy) showed that low irradiation doses (40, 50, and 60 Gy) did not significantly affect the insemination rate (Fig. 3, Additional file 1: Table S1). Similarly, insemination at 70 Gy (63.2%) did not differ significantly from the control (78.8%, *P* = 0.07; Additional file 1: Table S1). In contrast, intermediate doses (80 and 90 Gy) and all high doses (100, 110, and 120 Gy) significantly reduced insemination rates (Fig. 3, Additional file 1: Table S1).

### Egg hatching rate

**Fig. 4:**
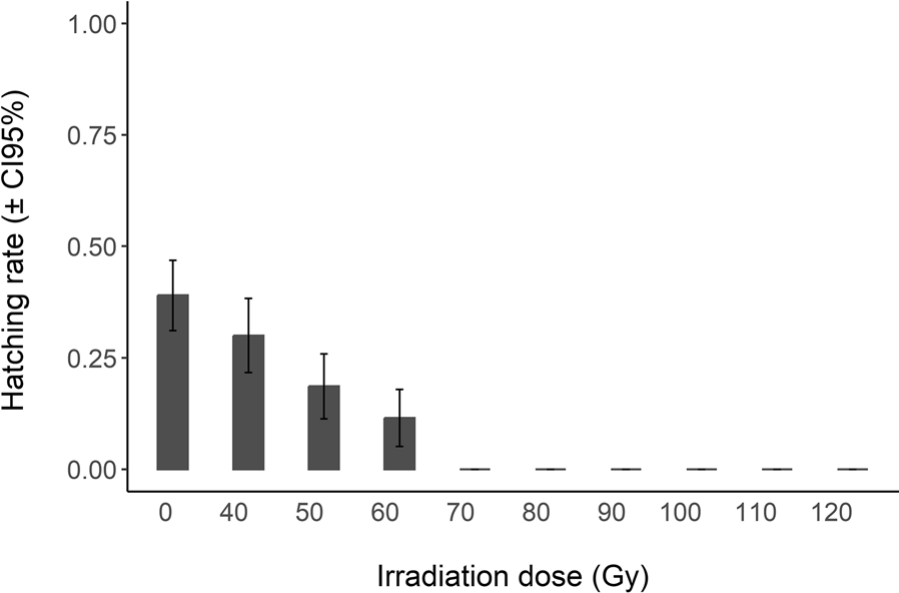
Hatching rate as a function of irradiation doses

Egg hatching rate was strongly affected by irradiation dose (Kruskal-Wallis *H* test: *χ*^2^ = 124.91*, df* = 9, *P* < *0.001*, Fig. 4). No significant differences were observed between the control and low-dose treatments, except at 60 Gy, which resulted in a significant reduction in hatching (Fig. 4, Additional file 2: Table S2). In contrast, hatching rates differed significantly between the control and all intermediate and high irradiation doses. Egg hatching was completely suppressed at doses of ≥ 70 Gy (Fig. 4).

## Discussion

The effectiveness of a sterile insect technique (SIT) program critically relies on the identification of an irradiation dose that induces high levels of sterility while minimizing adverse effects on key life-history traits [30]. In the present study, irradiation had no detectable effect on the adult emergence of *An.* *coluzzii* regardless of dose. This finding is consistent with previous studies reporting that irradiation does not impair pupal development or adult emergence in *Anopheles* species, including *An. arabiensis* [31, 36]. Similarly, studies conducted on *Aedes aegypti* and *Ae. albopictus* have yielded results similar to ours. Indeed, irradiation of these *Aedes* species at doses ranging from 15 to 90 Gy from a gamma source had no effect on pupal survival to adult emergence [37].

In contrast, mosquito survival was negatively affected by irradiation in a dose-dependent manner [38, 39]. All tested doses reduced longevity relative to controls, with the most pronounced effect observed at higher doses. These results align with previous reports documenting reduced survival in irradiated *Anopheles* mosquitoes, including *An.* *stephensi* and *An. pharoensis* [36, 40]. Similar effects have also been observed at high doses in *Ae. aegypti* and *Ae. albopictus* [37]*.* The observed reduction in longevity is likely attributable to radiation-induced somatic lesions, because although irradiation aims to induce dominant lethal mutations in germ cells, this process is not specific: somatic cells can be damaged [34, 40]. In fact, one of the most frequent effects of somatic lesions is reduced longevity [34]. However, the survival of male mosquitoes is important for the success of the sterile insect technique in the field. High longevity increases the probability that sterile males will encounter wild females and mate with them, thereby enhancing their sexual competitiveness and the operational impact of SIT programs [41, 42]. In this study, the survival rate of irradiated mosquitoes was lower than that of the control group, but we still obtained a median survival time of 12 days, ranging from 11 to 14 days, with a dose of 70 Gy. This duration was longer than the estimated median survival time of *Aedes* mosquitoes in the wild, which is 2 to 11 days for *Aedes aegypti* [43, 44] and 11 days for *Ae. albopictus* [44]. Although environmental factors may influence this survival time in the wild, this result could be sufficient to allow *An. coluzzii* mosquitoes irradiated at this dose to mate with wild females.

The insemination success was also influenced by the irradiation dose. While high (100, 110, and 120 Gy) and intermediate (80 and 90 Gy) doses significantly reduced insemination rates, low doses (40, 50, and 60 Gy) and the 70-Gy dose did not impair insemination compared with controls. This suggests that irradiation below a certain threshold does not adversely affect male mating ability, a finding consistent with earlier studies indicating that moderate irradiation doses preserve male sexual performance [31]. Similarly, a clear negative correlation has been observed between egg fertility and the dose of irradiation in several mosquito species [45–47].

Regarding fertility, low irradiation doses resulted in partial sterility, reflected by reduced but non-zero egg hatching rates. Conversely, irradiation at ≥ 70 Gy induced complete sterility in male mosquitoes, as evidenced by the absence of egg hatching. Similar results were observed by Bond et al. [37]. They found that in the *Ae. aegypti* population, the fertility of eggs from irradiated males mating with un-irradiated females was abolished at doses of 70 and 90 Gy [37]. This result also confirms the hypothesis that, beyond a critical dose threshold, dominant lethal mutations are induced in sperm, leading to the death of embryos in the early stages of development. Similar dose-dependent sterility thresholds have been reported in other *Anopheles* species [34, 38, 48, 49].

In light of these results, a 70-Gy dose could serve as a reference for *An. coluzzii* sterilization programs, achieving a zero hatching rate while preserving the ability of males to fertilize females. These results could support vector control strategies based on the sterile insect technique in endemic areas.

One limitation of our study lies in the method used to assess fecondity, namely insemination. The lack of quantitative parameters could affect the accuracy of our mosquito fecondity estimates. Nevertheless, we believe that fertility could be a crucial element of this study and potentially inform decision-making.

## Conclusions

This study evaluated the effects of different irradiation doses on key biological traits of *An. coluzzii* in the context of potential sterile insect technique implementation for malaria control in Africa. Our results demonstrate that male *An.* *coluzzii* can be effectively sterilized using gamma irradiation, with the optimal dose being 70 Gy. This dose induced complete male sterility without significantly affecting adult emergence or mating ability. Nevertheless, further research is needed to assess the sexual competitiveness of irradiated males compared to their wild counterparts, particularly under semi-natural and natural conditions. These studies will improve our understanding of the reproductive biology and behavior of *An. coluzzii* and will provide essential data for integrating SIT into malaria vector control strategies in Africa.

## Supplementary Information

Additional file 1.

Additional file 2.

## Data Availability

The data used in this article are available on request by contacting the corresponding authors.

## Abbreviations

%: Percent
cm: Centimeter
g: Gram
Gy: Gray
L: D: Light:dark
ml: Milliliter
mm: Millimeter
ºC: Degree Celsius
PCR: Polymerase chain reaction
RH: Relative humidity
s.l.: Sensu lato
SIT: Sterile insect technique

## References

[1] World Health Organization. World malaria report 2025. https://www.who.int/publications/i/item/9789240117822. Accessed 25 Feb 2026.

[2] Bhatt S, Weiss DJ, Cameron E, Bisanzio D, Mappin B, Dalrymple U, et al. The effect of malaria control on *Plasmodium falciparum* in Africa between 2000 and 2015. Nature. 2015;526:207–11. 10.1038/nature15535. 26375008 10.1038/nature15535 PMC4820050

[3] World Health Organization. World-malaria-report-2021-global-briefing-kit-eng. https://www.who.int/teams/global-malaria-programme/reports/world-malaria-report-2021. Accessed 26 Nov 2025.

[4] Alout H, Weill M. Amino-acid substitutions in acetylcholinesterase 1 involved in insecticide resistance in mosquitoes. Chem Biol Interact. 2008;175:138–41. 10.1016/j.cbi.2008.03.018. 18468592 10.1016/j.cbi.2008.03.018

[5] Ranson H, Abdallah H, Badolo A, Guelbeogo WM, Kerah-Hinzoumbé C, Yangalbé-Kalnoné E, et al. Insecticide resistance in *Anopheles gambiae*: data from the first year of a multi-country study highlight the extent of the problem. Malar J. 2009;8:299. 10.1186/1475-2875-8-299. 20015411 10.1186/1475-2875-8-299 PMC2804687

[6] Dabiré KR, Diabaté A, Namountougou M, Djogbenou L, Wondji C, Chandre F, et al. Trends in Insecticide Resistance in Natural Populations of Malaria Vectors in Burkina Faso, West Africa: 10 Years’ Surveys. Insectic - Pest Eng. InTech; 2012. p. 479–502. 10.5772/28749

[7] Namountougou M, Simard F, Baldet T, Diabaté A, Ouédraogo JB, Martin T, et al. Multiple insecticide resistance in *Anopheles gambiae* s.l. populations from Burkina Faso, West Africa. PLoS ONE. 2012;7:e48412. 10.1371/journal.pone.0048412. 23189131 10.1371/journal.pone.0048412 PMC3506617

[8] Churcher TS, Lissenden N, Griffin JT, Worrall E, Ranson H. The impact of pyrethroid resistance on the efficacy and effectiveness of bednets for malaria control in Africa. Elife. 2016;5:e16090. 10.7554/eLife.16090. 27547988 10.7554/eLife.16090 PMC5025277

[9] Alout H, Roche B, Dabiré RK, Cohuet A. Consequences of insecticide resistance on malaria transmission. PLoS Pathog. 2017;13:e1006499. 10.1371/journal.ppat.1006499. 28880906 10.1371/journal.ppat.1006499 PMC5589250

[10] Maxmen A. Scientists hail historic malaria vaccine approval but point to challenges ahead. Nature. 2021. 10.1038/d41586-021-02755-5. 34625728 10.1038/d41586-021-02755-5

[11] World Health Organization. WHO recommends groundbreaking malaria vaccine for children at risk 2021. https://www.who.int/fr/news/item/06-10-2021-who-recommends-groundbreaking-malaria-vaccine-for-children-at-risk. Accessed 2 Aug 2025.

[12] Datoo MS, Natama HM, Somé A, Bellamy D, Traoré O, Rouamba T, et al. Efficacy and immunogenicity of R21/Matrix-M vaccine against clinical malaria after 2 years’ follow-up in children in Burkina Faso: a phase 1/2b randomised controlled trial. Lancet Infect Dis. 2022. 10.1016/S1473-3099(22)00442-X. 36087586 10.1016/S1473-3099(22)00442-X

[13] Klassen W, Curtis CF. History of the sterile insect technique. In: Dyck VA, Hendrichs J, Robinson AS, editors. Sterile insect tech princ pract area-wide integr pest manag. Dordrecht: Springer; 2005. p. 3–36. 10.1007/1-4020-4051-2_1.

[14] Knipling EF. Possibilities of insect control or eradication through the use of sexually sterile males1. J Econ Entomol. 1955;48:459–62. 10.1093/jee/48.4.459.

[15] Bakri A, Mehta K, Lance DR. Sterilizing insects with Ionizing radiation. In: Sterile insect tech. 2nd ed. Boca Raton: CRC Press; 2021. p. 355–98. 10.1201/9781003035572-11.

[16] Bushland RC, Hopkins DE. Sterilization of screw-worm flies with x-rays and gamma-rays1. J Econ Entomol. 1953;46:648–56. 10.1093/jee/46.4.648.

[17] Lindquist AW. The use of gamma radiation for control or eradication of the screw-worm1. J Econ Entomol. 1955;48:467–9. 10.1093/jee/48.4.467.

[18] Lofgren CS, Dame DA, Breeland SG, Weidhaas DE, Jeffery G, Kaiser R, et al. Release of chemosterilized males for the control of *Anopheles**albimanus* in El Salvador: III. Field methods and population control. Am J Trop Med Hyg. 1974;23:288–97. 10.4269/ajtmh.1974.23.288. 4817674 10.4269/ajtmh.1974.23.288

[19] Knipling EF. Sterile-male method of population control: Successful with some insects, the method may also be effective when applied to other noxious animals. Science. 1959;130:902–4. 10.1126/science.130.3380.902. 14410136 10.1126/science.130.3380.902

[20] Hendrichs J, Dyck AA, Robinson AS. Sterile insect technique: principles and practice in area-wide integrated pest managment. Dordrecht: Springer; 2005.

[21] Dyck VA, Hendrichs J, Robinson AS. Sterile insect technique: principles and practice in area-wide integrated pest management. Dordrecht, Netherlands: Springer; 2005.

[22] Vreysen MJ, Saleh KM, Ali MY, Abdulla AM, Zhu ZR, Juma KG, et al. *Glossina austeni* (*Diptera: Glossinidae*) eradicated on the island of Unguja, Zanzibar, using the sterile insect technique. J Econ Entomol. 2000;93:123–35. 10.1603/0022-0493-93.1.123. 14658522 10.1603/0022-0493-93.1.123

[23] Bellini R, Medici A, Puggioli A, Balestrino F, Carrieri M. Pilot field trials with *Aedes albopictus* irradiated sterile males in Italian urban areas. J Med Entomol. 2013;50:317–25. 10.1603/ME12048. 23540120 10.1603/me12048

[24] Benedict MQ, Robinson AS. The first releases of transgenic mosquitoes: an argument for the sterile insect technique. Trends Parasitol. 2003;19:349–55. 10.1016/s1471-4922(03)00144-2. 12901936 10.1016/s1471-4922(03)00144-2

[25] Bassène MD, Seck MT, Pagabeleguem S, Fall AG, Sall B, Vreysen MJB, et al. Competitiveness and survival of two strains of *Glossina palpalis gambiensis* in an urban area of Senegal. PLoS Negl Trop Dis. 2017;11:e0006172. 10.1371/journal.pntd.0006172. 29281634 10.1371/journal.pntd.0006172 PMC5760099

[26] Benelli G. Research in mosquito control: current challenges for a brighter future. Parasitol Res. 2015;114:2801–5. 10.1007/s00436-015-4586-9. 26093499 10.1007/s00436-015-4586-9

[27] Reisen WK, Baker RH, Sakai RK, Mahmood F, Rathor HR, Raana K, et al. *Anopheles culicifacies* Giles:1 mating behavior and competitiveness in nature of chemosterilized males carrying a genetic sexing system. Ann Entomol Soc Am. 1981;74:395–401. 10.1093/aesa/74.4.395.

[28] Maiga H. Etude de la bio-écologie des mâles d’*Anopheles gambiae* s.l. et optimisation des méthodes d’élevage dans une perspective de développement de la lutte génétique. Thèse de Doctorat à l’Université Polytechnique de Bobo-Dioulasso, 144P. 2015. 10.13140/RG.2.2.14982.63049

[29] Poda SB, Guissou E, Maïga H, Bimbile-Somda SN, Gilles J, Rayaisse J-B, et al. Impact of irradiation on the reproductive traits of field and laboratory *An. arabiensis* mosquitoes. Parasit Vectors. 2018;11:641. 10.1186/s13071-018-3228-3. 30558681 10.1186/s13071-018-3228-3 PMC6296153

[30] Helinski ME, Parker AG, Knols BG. Radiation biology of mosquitoes. Malar J. 2009;8:S6. 10.1186/1475-2875-8-S2-S6. 19917076 10.1186/1475-2875-8-S2-S6 PMC2777328

[31] Helinski MEH, Knols BGJ. The influence of late-stage pupal irradiation and increased irradiated: un-irradiated male ratio on mating competitiveness of the malaria mosquito *Anopheles arabiensis*. Bull Entomol Res. 2009;99:317–22. 10.1017/S0007485308006354. 19063756 10.1017/S0007485308006354

[32] Bakri A, Heather N, Hendrichs J, Ferris I. Fifty years of radiation biology in entomology. Ann Entomol Soc Am. 2005;98:1–12.

[33] Hallman GJ. Ionizing irradiation quarantine treatment against plum curculio (*Coleoptera: Curculionidae*). J Econ Entomol. 2003;96:1399–404. 10.1093/jee/96.5.1399. 14650511 10.1603/0022-0493-96.5.1399

[34] Helinski ME, Parker AG, Knols BG. Radiation-induced sterility for pupal and adult stages of the malaria mosquito *Anopheles arabiensis*. Malar J. 2006;5:41. 10.1186/1475-2875-5-41. 16700906 10.1186/1475-2875-5-41 PMC1475870

[35] R Core Team. A language and environment for statistical computing (4.5.1) [Computer software]. 2025. https://cran.r-project.org/src/base/R-4

[36] Munhenga G, Brooke BD, Gilles JRL, Slabbert K, Kemp A, Dandalo LC, et al. Mating competitiveness of sterile genetic sexing strain males (GAMA) under laboratory and semi-field conditions: steps towards the use of the Sterile Insect Technique to control the major malaria vector *Anopheles arabiensis* in South Africa. Parasit Vectors. 2016;9:122. 10.1186/s13071-016-1385-9. 26934869 10.1186/s13071-016-1385-9 PMC4774148

[37] Bond JG, Osorio AR, Avila N, Gómez-Simuta Y, Marina CF, Fernández-Salas I, et al. Optimization of irradiation dose to *Aedes aegypti* and *Ae. albopictus* in a sterile insect technique program. PLoS ONE. 2019;14:e0212520. 10.1371/journal.pone.0212520. 30779779 10.1371/journal.pone.0212520 PMC6380561

[38] Balestrino F, Puggioli A, Carrieri M, Bouyer J, Bellini R. Quality control methods for *Aedes albopictus* sterile male production. PLoS Negl Trop Dis. 2017;11:e0005881. 10.1371/journal.pntd.0005881. 28892483 10.1371/journal.pntd.0005881 PMC5608434

[39] Ranathunge T, Harishchandra J, Maiga H, Bouyer J, Gunawardena YINS, Hapugoda M. Development of the Sterile Insect Technique to control the dengue vector *Aedes aegypti* (*Linnaeus*) in Sri Lanka. PLoS ONE. 2022;17:e0265244. 10.1371/journal.pone.0265244. 35377897 10.1371/journal.pone.0265244 PMC8979456

[40] Sharma VP, Razdan RK, Ansari MA. *Anopheles stephensi*: effect of gamma-radiation and chemosterilants on the fertility and fitness of males for sterile male releases13. J Econ Entomol. 1978;71:449–52. 10.1093/jee/71.3.449. 690317 10.1093/jee/71.3.449

[41] Harris AF, McKemey AR, Nimmo D, Curtis Z, Black I, Morgan SA, et al. Successful suppression of a field mosquito population by sustained release of engineered male mosquitoes. Nat Biotechnol. 2012;30:828–30. 10.1038/nbt.2350. 22965050 10.1038/nbt.2350

[42] Bouyer J, Culbert NJ, Dicko AH, Pacheco MG, Virginio J, Pedrosa MC, et al. Field performance of sterile male mosquitoes released from an uncrewed aerial vehicle. Sci Robot. 2020;5:eaba6251. 10.1126/scirobotics.aba6251. 33022616 10.1126/scirobotics.aba6251

[43] Sheppard PM, Macdonald WW, Tonn RJ, Grab B. The dynamics of an adult population of *Aedes aegypti* in relation to Dengue Haemorrhagic Fever in Bangkok. J Anim Ecol. 1969;38:661. 10.2307/3042.

[44] Brady OJ, Johansson MA, Guerra CA, Bhatt S, Golding N, Pigott DM, et al. Modelling adult *Aedes aegypti* and *Aedes albopictus s*urvival at different temperatures in laboratory and field settings. Parasit Vectors. 2013;6:351. 10.1186/1756-3305-6-351. 24330720 10.1186/1756-3305-6-351 PMC3867219

[45] Balestrino F, Medici A, Candini G, Carrieri M, Maccagnani B, Calvitti M, et al. Gamma ray dosimetry and mating capacity studies in the laboratory on *Aedes albopictus* males. J Med Entomol. 2010;47:581–91. 10.1603/me09272. 20695273 10.1093/jmedent/47.4.581 PMC7027263

[46] Shetty V, Shetty NJ, Harini BP, Ananthanarayana SR, Jha SK, Chaubey RC. Effect of gamma radiation on life history traits of *Aedes aegypti* (L.). Parasite Epidemiol Control. 2016;1:26–35. 10.1016/j.parepi.2016.02.007. 29988174 10.1016/j.parepi.2016.02.007 PMC5991819

[47] Oliva CF, Jacquet M, Gilles J, Lemperiere G, Maquart P-O, Quilici S, et al. The sterile insect technique for controlling populations of *Aedes albopictus (Diptera: Culicidae)* on reunion island: mating Vigour of sterilized males. PLoS ONE. 2012;7:e49414. 10.1371/journal.pone.0049414. 23185329 10.1371/journal.pone.0049414 PMC3504010

[48] Robinson AS. Genetic Basis of the Sterile Insect Technique. Sterile Insect Tech. 2nd ed. Boca Raton: CRC Press; 2021. p. 143–62. 10.1201/9781003035572-5

[49] Chen C, Aldridge RL, Gibson S, Kline J, Aryaprema V, Qualls W, et al. Developing the radiation-based sterile insect technique (SIT) for controlling *Aedes aegypti*: identification of a sterilizing dose. Pest Manag Sci. 2023;79:1175–83. 10.1002/ps.7303. 36424673 10.1002/ps.7303

