# Supplementary material for: Optimization of the irradiation dose for Anopheles coluzzii for implementation of the sterile insect technique
Source: Parasit Vectors. 2026 May 5;19:261. doi: 10.1186/s13071-026-07396-z (PMC13289122; doi:10.1186/s13071-026-07396-z)
Supplement: Supplementary file 1 — Additional file 1. [file 13071_2026_7396_MOESM1_ESM.docx]

| Doses | | diff | | lwr | | upr | | P.adj | |
| --- | --- | --- | --- | --- | --- | --- | --- | --- | --- |
| 40 – 0 | 0.018 | | 0.182 | | 0.146 | | 1.000 | |  |
| 50 – 0 | 0.072 | | 0.236 | | 0.092 | | 0.906 | |  |
| 60 – 0 | 0.110 | | 0.274 | | 0.054 | | 0.456 | |  |
| 70 – 0 | 0.157 | | 0.321 | | 0.007 | | 0.072 | |  |
| 80 – 0 | 0.207 | | 0.371 | | 0.043 | | 0.004 | |  |
| 90 – 0 | 0.250 | | 0.414 | | 0.086 | | 0.000 | |  |
| 100 – 0 | 0.330 | | 0.494 | | 0.166 | | 0.000 | |  |
| 110 – 0 | 0.357 | | 0.521 | | 0.193 | | 0.000 | |  |
| 120 – 0 | 0.400 | | 0.564 | | 0.236 | | 0.000 | |  |
| 50 – 40 | 0.053 | | 0.217 | | 0.111 | | 0.985 | |  |
| 60 – 40 | 0.092 | | 0.256 | | 0.072 | | 0.700 | |  |
| 70 – 40 | 0.138 | | 0.302 | | 0.026 | | 0.167 | |  |
| 80 – 40 | 0.188 | | 0.352 | | 0.024 | | 0.013 | |  |
| 90 – 40 | 0.232 | | 0.396 | | 0.068 | | 0.001 | |  |
| 60 – 50 | 0.038 | | 0.202 | | 0.126 | | 0.999 | |  |
| 70 – 50 | 0.085 | | 0.249 | | 0.079 | | 0.781 | |  |
| 80 – 50 | 0.135 | | 0.299 | | 0.029 | | 0.191 | |  |
| 90 – 50 | 0.178 | | 0.342 | | 0.014 | | 0.023 | |  |
| 70 – 60 | 0.047 | | 0.211 | | 0.117 | | 0.994 | |  |
| 80 – 60 | 0.097 | | 0.261 | | 0.067 | | 0.635 | |  |
| 90 – 60 | 0.140 | | 0.304 | | 0.024 | | 0.155 | |  |
| 80 – 70 | 0.050 | | 0.214 | | 0.114 | | 0.990 | |  |
| 90 – 70 | 0.093 | | 0.257 | | 0.071 | | 0.679 | |  |
| 90 – 80 | 0.043 | | 0.207 | | 0.121 | | 0.997 | |  |
| 40 – 100 | 0.312 | | 0.148 | | 0.476 | | 0.000 | |  |
| 50 – 100 | 0.258 | | 0.094 | | 0.422 | | 0.000 | |  |
| 60 – 100 | 0.220 | | 0.056 | | 0.384 | | 0.002 | |  |
| 70 – 100 | 0.173 | | 0.009 | | 0.337 | | 0.030 | |  |
| 80 – 100 | 0.123 | | 0.041 | | 0.287 | | 0.298 | |  |
| 90 – 100 | 0.080 | | 0.084 | | 0.244 | | 0.834 | |  |
| 110 – 100 | 0.027 | | 0.191 | | 0.137 | | 1.000 | |  |
| 120 – 100 | 0.070 | | 0.234 | | 0.094 | | 0.917 | |  |
| 40 – 110 | 0.338 | | 0.174 | | 0.502 | | 0.000 | |  |
| 50 – 110 | 0.285 | | 0.121 | | 0.449 | | 0.000 | |  |
| 60 – 110 | 0.247 | | 0.083 | | 0.411 | | 0.000 | |  |
| 70 – 110 | 0.200 | | 0.036 | | 0.364 | | 0.006 | |  |
| 80 – 110 | 0.150 | | 0.014 | | 0.314 | | 0.099 | |  |
| 90 – 110 | 0.107 | | 0.057 | | 0.271 | | 0.500 | |  |
| 120 – 110 | 0.043 | | 0.207 | | 0.121 | | 0.997 | |  |
| 40 – 120 | 0.382 | | 0.218 | | 0.546 | | 0.000 | |  |
| 50 – 120 | 0.328 | | 0.164 | | 0.492 | | 0.000 | |  |
| 60 – 120 | 0.290 | | 0.126 | | 0.454 | | 0.000 | |  |
| 70 – 120 | 0.243 | | 0.079 | | 0.407 | | 0.000 | |  |
| 80 – 120 | 0.193 | | 0.029 | | 0.357 | | 0.010 | |  |
| 90 – 120 | 0.150 | | 0.014 | | 0.314 | | 0.099 | |  |
