# Supplementary material for: Optimization of the irradiation dose for Anopheles coluzzii for implementation of the sterile insect technique
Source: Parasit Vectors. 2026 May 5;19:261. doi: 10.1186/s13071-026-07396-z (PMC13289122; doi:10.1186/s13071-026-07396-z)
Supplement: Supplementary file 2 — Additional file 2. [file 13071_2026_7396_MOESM2_ESM.docx]

| Dose |  | Dose | Z | P.unadj | P.adj |
| --- | --- | --- | --- | --- | --- |
| 40 |  | 0 | 1.199 | 2.30e-01 | 1.00e+00 |
| 50 |  | 0 | 2.109 | 3.49e-02 | 1.00e+00 |
| 60 |  | 0 | 2.988 | 2.81e-03 | 1.26e-01 |
| 70 |  | 0 | 6.209 | 5.32e-10 | 2.39e-08 |
| 80 |  | 0 | 5.882 | 4.05e-09 | 1.82e-07 |
| 90 |  | 0 | 6.276 | 3.48e-10 | 1.57e-08 |
| 100 |  | 0 | 6.209 | 5.32e-10 | 2.39e-08 |
| 110 |  | 0 | 5.975 | 2.30e-09 | 1.04e-07 |
| 120 |  | 0 | 6.395 | 1.61e-10 | 7.24e-09 |
| 50 |  | 40 | 1.253 | 2.10e-01 | 1.00e+00 |
| 60 |  | 40 | 2.306 | 2.11e-02 | 9.50e-01 |
| 70 |  | 40 | 6.124 | 9.14e-10 | 4.12e-08 |
| 80 |  | 40 | 5.671 | 1.42e-08 | 6.40e-07 |
| 90 |  | 40 | 6.220 | 4.99e-10 | 2.24e-08 |
| 60 |  | 50 | 1.035 | 3.01e-01 | 1.00e+00 |
| 70 |  | 50 | 3.899 | 9.67e-05 | 4.35e-03 |
| 80 |  | 50 | 3.693 | 2.21e-04 | 9.96e-03 |
| 90 |  | 50 | 3.940 | 8.14e-05 | 3.66e-03 |
| 70 |  | 60 | 2.441 | 1.46e-02 | 6.59e-01 |
| 80 |  | 60 | 2.333 | 1.97e-02 | 8.85e-01 |
| 90 |  | 60 | 2.463 | 1.38e-02 | 6.20e-01 |
| 80 |  | 70 | 0.000 | 1.00e+00 | 1.00e+00 |
| 90 |  | 70 | 0.000 | 1.00e+00 | 1.00e+00 |
| 90 |  | 80 | 0.000 | 1.00e+00 | 1.00e+00 |
| 40 |  | 100 | -6.124 | 9.14e-10 | 4.12e-08 |
| 50 |  | 100 | -3.899 | 9.67e-05 | 4.35e-03 |
| 60 |  | 100 | -2.441 | 1.46e-02 | 6.59e-01 |
| 70 |  | 100 | 0.000 | 1.00e+00 | 1.00e+00 |
| 80 |  | 100 | 0.000 | 1.00e+00 | 1.00e+00 |
| 90 |  | 100 | 0.000 | 1.00e+00 | 1.00e+00 |
| 110 |  | 100 | 0.000 | 1.00e+00 | 1.00e+00 |
| 120 |  | 100 | 0.000 | 1.00e+00 | 1.00e+00 |
| 40 |  | 110 | -5.796 | 6.79e-09 | 3.06e-07 |
| 50 |  | 110 | -3.751 | 1.76e-04 | 7.91e-03 |
| 60 |  | 110 | -2.364 | 1.81e-02 | 8.14e-01 |
| 70 |  | 110 | 0.000 | 1.00e+00 | 1.00e+00 |
| 80 |  | 110 | 0.000 | 1.00e+00 | 1.00e+00 |
| 90 |  | 110 | 0.000 | 1.00e+00 | 1.00e+00 |
| 120 |  | 110 | 0.000 | 1.00e+00 | 1.00e+00 |
| 40 |  | 120 | -6.395 | 1.61e-10 | 7.22e-09 |
| 50 |  | 120 | -4.015 | 5.94e-05 | 2.68e-03 |
| 60 |  | 120 | -2.501 | 1.24e-02 | 5.57e-01 |
| 70 |  | 120 | 0.000 | 1.00e+00 | 1.00e+00 |
| 80 |  | 120 | 0.000 | 1.00e+00 | 1.00e+00 |
| 90 |  | 120 | 0.000 | 1.00e+00 | 1.00e+00 |
